# Genetic Analysis of Polyunsaturated Fatty Acids Biosynthesis Pathway Determines Four Distinct Thraustochytrid Types

**DOI:** 10.1111/1462-2920.70090

**Published:** 2025-03-28

**Authors:** Sou‐Yu Cheng, Yi‐Jing Chen, Hsiu‐Chin Lin, Hsin‐Yang Chang, Ming‐Der Huang

**Affiliations:** ^1^ Department of Biological Sciences National Sun Yat‐sen University Kaohsiung Taiwan; ^2^ Department of Marine Biotechnology and Resources National Sun Yat‐sen University Kaohsiung Taiwan; ^3^ Department of Life Sciences and Institute of Genome Sciences National Yang Ming Chiao Tung University Taipei Taiwan

**Keywords:** β‐carotene hydroxylase, ATP‐citrate lyase, carotenoids, polyunsaturated fatty acids (PUFAs), PUFA biosynthesis pathways, thraustochytrids

## Abstract

Thraustochytrids, diverse marine unicellular protists encompassing over 10 recognised genera, are renowned for synthesising polyunsaturated fatty acids (PUFAs), with content and composition varying substantially across genera. While PUFAs are known to be produced via PUFA synthase (PUFA‐S) and/or elongase/desaturase (ELO/DES) pathways, the distinctions in genes involved remain unexplored. This study analysed PUFA biosynthetic genes in 19 thraustochytrid strains across six genera, categorising them into four types. Type I exclusively utilises the ELO/DES pathway, Type II employs both PUFA‐S and complete ELO/DES pathways, while Types III and IV primarily rely on PUFA‐S, with Type III lacking the canonical Δ9 desaturase and Type IV missing most desaturase and elongase enzymes. Notably, the Δ9 desaturase and ATP‐citrate lyase (*ACLY*) are exclusive to Types I and II, while β‐carotene hydroxylase (*CrtZ*) is absent in these types. ACLY absence suggests alternative acetyl‐CoA supply pathways in Types III and IV, whereas CrtZ absence implies either a lack of specific xanthophylls or alternative biosynthetic pathways in Types I and II. Synteny analysis revealed conserved genomic organisation of PUFA biosynthetic genes, indicating a shared evolutionary trajectory. This study provides insights into the genetic diversity underlying PUFA biosynthesis in thraustochytrids, while proposing putative evolutionary pathways for the four lineages.

## Graphical abstract

Thraustochytrids exhibit four distinct polyunsaturated fatty acid (PUFA) biosynthetic types: Type I (ELO/DES pathway), Type II (dual‐pathway) and Types III–IV (PUFA‐S pathway with varying gene losses). This genetic diversity in PUFA biosynthesis pathways provides new perspectives for understanding their evolutionary relationships and potential taxonomic classification.

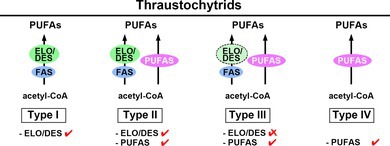

## 1 Introduction

Thraustochytrids are a group of osmoheterotrophic marine protists distinguished by their remarkable biotechnological potential. These microorganisms are renowned for producing diverse enzymes with significant industrial applications and accumulating valuable metabolites like polyunsaturated fatty acids (PUFAs), carotenoids and squalene derivatives (Aasen et al. 2016; Lin et al. 2020). Taxonomically, thraustochytrids have a complex classification history. Initially misclassified as primitive fungi and subsequently as Oomycetes due to their rhizoid‐like ectoplasmic nets (EN) and biflagellate zoospores, they are now positioned within the Order Thraustochytrida, subclass Labyrinthulomycetes, in the kingdom Chromista as plastid‐free heterokont algae (Ellenbogen et al. 1969; Cavalier‐Smith et al. 1994; Tsui et al. 2009; Anderson and Cavalier‐Smith 2012). For further classification, thraustochytrid strains from different habitats worldwide were categorised into different genera by their distinct life cycle styles (Bremer 1976; Honda et al. 1998; Bongiorni et al. 2005; Iwata et al. 2017). However, this genus determination method became controversial since identical thraustochytrid genera can display different lifestyles and morphological traits depending on their environmental conditions (Dellero, Rose, et al. 2018; Dellero, Cagnac, et al. 2018). This prompted scientists to refine classification methods by incorporating phylogenetic evidence from 5S and 18S rRNA along with lifecycle morphologies, phylogenomic analysis and biochemical profiles, such as PUFA and carotenoids compositions, within thraustochytrids, resulting in the establishment of 13 thraustochytrid genera (Huang et al. 2003; Yokoyama and Honda 2007; Yokoyama et al. 2007; Morabito et al. 2019; Hassett 2020; Geraci‐Yee et al. 2021).

The cell morphologies of thraustochytrids are intricately linked to their distinct life cycle stages and genera. The life cycles of the three most productive genera for their total fatty acid and docosahexaenoic acid (DHA) production, namely *Aurantiochytrium*, *Thraustochytrium* and *Schizochytrium*, have been particularly well‐studied (Bongiorni et al. 2005; Yokoyama et al. 2007; Ganuza et al. 2019). Typically, their life cycles commence with diploid mononucleated cells, which either undergo several rounds of karyokinesis to form multinucleated cells (*Aurantiochytrium* and *Thraustochytrium*), or successive bipartition to increase cell numbers and form cell clusters (*Aurantiochytrium* and *Schizochytrium*); lipid accumulation is initiated at this stage in response to environmental cues. In the former scenario, cytokinesis occurs later in the multinucleated cells to generate sporangia, from which encompassing biflagellate zoospores are released to seek new habitats, settle, and mature into mononucleated cells, restarting the cycle. In the latter case, each cell in the cell cluster also generates into a sporangium to release zoospores (Dellero, Rose, et al. 2018; Fossier Marchan et al. 2018; Morabito et al. 2019). In addition, motile amoeboid cells have been observed in the life cycles of several thraustochytrid genera, including *Aurantiochytrium*, *Thraustochytrium*, *Ulkenia*, and its derived genera *Botryochytrium* and *Parietichytrium* (Raghukumar 1992; Honda et al. 1998; Yokoyama et al. 2007). Inhabiting primarily in nutrient‐rich sediments, mononucleated thraustochytrids halt cell division and initiate triacylglycerol (TAG) accumulation when the nitrogen source is depleted and the carbon source remains sufficient. The carbon flux intended for constructing new cell components is diverted solely toward fatty acid synthesis. One proposed mechanism is that nitrogen limitation leads to low AMP levels, which disrupt the tricarboxylic acid (TCA) cycle, causing citrate accumulation and transport to the cytosol as a substrate for ATP‐citrate lyase (ACLY) to produce acetyl‐CoA for de novo fatty acid synthesis and subsequent TAG accumulation (Ratledge 2004; Ren et al. 2009; Chaisawang et al. 2012). The accumulated TAG is dispersed into each single cell in sporangia to fuel zoospore or amoeboid cell movement and re‐habitation.

Unlike most oleaginous microorganisms, where PUFAs are present only in membrane lipids, thraustochytrids produce long chain ω3‐PUFAs, including DHA (C22:6, n‐3) and eicosapentaenoic acid (EPA, C20:5, n‐3), as major portions within storage lipids such as TAG (Goold et al. 2015; Xie and Wang 2015). There are two pathways operating in thraustochytrids to produce PUFAs: the elongase/desaturase (ELO/DES) pathway and the PUFA synthase (PUFA‐S) pathway. The ELO/DES pathway utilises the conventional fatty acid synthase (FAS) to produce palmitic acid (C16:0), which is then further elongated and desaturated into C16–C22 PUFAs through specific elongases and desaturases (Qiu 2003). The desaturation process in this pathway requires oxygen consumption; consequently, unsaturated C16 and C18 fatty acids cannot be detected in thraustochytrids under anaerobic conditions (Jakobsen et al. 2008). The PUFA‐S pathway involves a unique enzymatic system specially designed for EPA or DHA synthesis. It employs a polyketide synthase (PKS)‐like multi‐subunit protein complex comprising PfaA, PfaB and PfaC subunits. This complex produces C20–C22 unsaturated fatty acids through successive malonyl‐CoA‐dependent elongation steps, utilising isomerization rather than double bond removal, thus eliminating the need for oxygen‐dependent desaturation (Meesapyodsuk and Qiu 2016; Qiu et al. 2020).

While the distribution of PUFA biosynthesis pathways varies among thraustochytrid species, with some possessing both ELO/DES and PUFA‐S systems and others harbouring only one (Lippmeier et al. 2009; Ishibashi et al. 2021), these pathways are not functionally redundant. Lippmeier et al. (2009) demonstrated this non‐redundancy when mutants with inactive PUFA‐S became auxotrophs, requiring external PUFA supplementation for survival. Further evidence for the distinct roles of these pathways comes from knockout studies, where disrupting either Δ5 elongase in the ELO/DES pathway or the PfaC subunit in the PUFA‐S pathway severely reduced cell growth in *Aurantiochytrium* sp. (Liang et al. 2018). Interestingly, some thraustochytrids possess a complete PUFA‐S pathway alongside a defective ELO/DES pathway (Meesapyodsuk and Qiu 2016). Studies of *Aurantiochytrium limacinum* and *Schizochytrium* sp. suggest that these organisms contain an incomplete ELO/DES pathway for PUFA biosynthesis, which could potentially function in retrieving substrates from the environment or salvaging intermediates from pathway leakages (Lippmeier et al. 2009; Kobayashi et al. 2011).

To better understand the distribution and conservation of these two PUFA biosynthetic routes across thraustochytrid genera and species, and to evaluate their potential as markers for taxonomic classification, we conducted a comprehensive comparative analysis of genomic and transcriptomic data from 19 thraustochytrid strains representing six genera. Our analysis revealed that most strains possess either a complete ELO/DES pathway or a PUFA‐S pathway, but rarely both. Based on pathway‐specific gene patterns, we categorised these strains into four distinct groups. Notably, the gene encoding ACLY was exclusive to strains with a complete ELO/DES pathway, while one for β‐carotene hydroxylase (*CrtZ*) appeared only in strains lacking a complete ELO/DES pathway. Synteny analysis further revealed conserved genomic contexts for PUFA biosynthesis‐related genes. Based on these findings, we proposed potential evolutionary trajectories among the four thraustochytrid lineages.

## 2 Material and Methods

### 2.1 Gene Identification From Genomic Databases

Genomic databases were obtained from the National Center for Biotechnology Information (NCBI, https://www.ncbi.nlm.nih.gov/) or the MycoCosm database of Joint Genome Institute (JGI) website (https://mycocosm.jgi.doe.gov/; Table 1 and Table S1). The database from *Hondaea fermentalgiana*, with its more comprehensive and well‐annotated protein database, was initially utilised for target gene identification, and the acquired sequence served as the closer template sequences for homologues in other thraustochytrids. Gene identification was performed by utilising sequence similarity searches with the BLAST program and motif searches with the HMMER program (Potter et al. 2018). The sequence identification process was initiated with keyword searches using the NCBI engine for putative protein sequences of interest in all investigated thraustochytrids and their closely relative species. These collected sequences were then used as queries for homologous sequence identification against genomic and protein databases of the target thraustochytrid strains using the BLAST program. Concurrently, protein candidates with potential conserved motifs were selected using the HMMER program with default settings; the generated Pfam IDs are provided in the results section. Subsequently, the collected candidate sequences were analysed by the FGENESH+ program on the Softberry website for validation of transcript and protein sequences, with parameters set for the model organisms *Saccharomyces cerevisiae* , *Chlamydomonas reinhardtii* and *Arthrobotrys oligospora* as templates (http://www.softberry.com). Putative protein sequences of the candidates were further validated using the Motif Search tool provided by GenomeNet (https://www.genome.jp/tools/motif/) based on the hidden Markov model (HMM) to identify their distinctive protein features.

**TABLE 1:** Detail of the sequenced thraustochytrid genomes used in this study.

Species	Order	Family	Strain	Accession number	Database
Type I					
*Parietichytrium* sp. I65‐24A	Thraustochytrida	Thraustochytriaceae	I65‐24A	GCA_012862575.1	NCBI
*Schizochytrium aggregatum*	Thraustochytrida	Thraustochytriaceae	ATCC 28209	Project ID: 402022	JGI
*Thraustochytrium striatum*	Thraustochytrida	Thraustochytriaceae	ATCC 24473	GCA_022244645.1	NCBI
Type II					
*Thraustochytrium aureum*	Thraustochytrida	Thraustochytriaceae	ATCC 34304	GCA_012862495.1	NCBI
Type III					
*Aurantiochytrium* sp. T66	Thraustochytrida	Thraustochytriaceae	T66	GCA_001462505.1	NCBI
*Aurantiochytrium* sp. KH105	Thraustochytrida	Thraustochytriaceae	KH105	GCA_003116975.1	NCBI
*Hondaea fermentalgiana*	Thraustochytrida	Thraustochytriaceae	FCC1311	GCA_014084085.1	NCBI
*Thraustochytrium* sp. ATCC 26185	Thraustochytrida	Thraustochytriaceae	ATCC 26185	GCA_002154235.1	NCBI
*Thraustochytrium* sp. TN22	Thraustochytrida	Thraustochytriaceae	TN22	GCA_013306675.1	NCBI
*Schizochytrium* sp. CCTCC M209059	Thraustochytrida	Thraustochytriaceae	CCTCC M209059	GCA_000818945.1	NCBI
Type IV					
*Aurantiochytrium acetophilum*	Thraustochytrida	Thraustochytriaceae	HS399	GCA_004332575.1	NCBI
*Aurantiochytrium limacinum*	Thraustochytrida	Thraustochytriaceae	ATCC MYA‐1381	Project ID: 1450242	JGI
*Aurantiochytrium limacinum*	Thraustochytrida	Thraustochytriaceae	SR21	GWHABLD00000000	NGDC
*Schizochytrium* sp. TIO01	Thraustochytrida	Thraustochytriaceae	TIO01	GCA_004764695.1	NCBI
Close relatives					
*Aplanochytrium kerguelense*	Labyrinthulida	Aplanochytriaceae	PBS07	Project ID: 402023	JGI
*Labyrinthula* sp. SR_Ha_C	Labyrinthulida	Labyrinthulaceae	SR_Ha_C	GCA_015227615.1	JGI

*Note:* The genomic databases of 14 thraustochytrids and two close relatives were downloaded from the NCBI (National Center for Biotechnology Information), JGI (DOE Joint Genome Institute) and NGDC (National Genomics Data Center) databases.

The identified sequences in *H. fermentalgiana* were then used as queries for a second round of target gene identification in the database of other thraustochytrid strains using the procedures described above. For strains with only genomic databases available but lacking protein databases, putative protein sequences were predicted and acquired using the ab initio prediction program Augustus, with parameters set for the model organisms *S. cerevisiae* and *C. reinhardtii* . Finally, all putative protein sequences belonging to the same enzyme family underwent multiple sequence alignment using the MUSCLE program for further sequence refinement. Any sequences with erroneous predictions were manually corrected. Protein sequences, comprising both publicly available and newly predicted sequences from genomic and RNA‐seq databases, are detailed in Table S2.

### 2.2 Gene Identification From Transcriptomic Databases

Raw transcriptomic data (RNA‐seq) were retrieved from the Sequence Read Archive (SRA) hosted by NCBI (https://www.ncbi.nlm.nih.gov/sra; Table S3). Sequence reads were extracted using the fastq‐dump algorithm provided by the SRA‐Toolkit (https://github.com/ncbi/sra‐tools). De novo assembly of the reads were performed using the Trinity program with default settings to generate assembled transcriptome sequences (Haas et al. 2013). Following the assembly, transcript sequences underwent analysis with the TransDecoder program (http://transdecoder.sf.net/) to identify the transcript and protein sequences with long open reading frame (ORF) and the most likely coding regions. Both the transcriptome and protein databases were then utilised for gene identification, employing procedures similar to those described in the previous section for genomic databases, including sequence similarity searches, motif analyses, and multiple sequence alignments.

### 2.3 Multiple Sequence Alignment and Phylogenetic Tree Construction

Phylogenetic trees were constructed using either protein or DNA sequences. Prior to tree construction, multiple sequence alignments were performed using the MAFFT program (Katoh et al. 2019). Phylogenetic trees and bootstrap values were generated using tools from the PHYLIP package (Retief 2000). For trees constructed with putative protein sequences, protein distances were estimated using the PROTDIST tool with aligned sequence files. The neighbour‐joining (NJ) method from the PHYLIP package was then used to construct the trees. For trees constructed with 18S rRNA gene sequences, sequences were acquired from the NCBI GeneBank database or identified from the investigated genomic databases (Table S4). The DNA sequences were aligned using the MAFFT program. Tree generation was performed using the Maximum Likelihood method with the DNAML tool within the PHYLIP package. Bootstrap support values were calculated using the SEQBOOT tool to generate 1000 bootstrap replicates, and the BOOSTER program was employed to compute the values on each branch of the inferred phylogenetic trees (Lemoine et al. 2018). Finally, all trees were visualised using the online program iTOL (https://itol.embl.de/).

### 2.4 Synteny Analysis

Synteny analysis was performed using approximately four genes located upstream and downstream of the target gene. Prior to the synteny analysis, gene prediction was conducted on the flanking regions of the target gene. Initially, ORFs longer than 60 amino acids were identified using the ORFfinder program (https://www.ncbi.nlm.nih.gov/orffinder/). The BLASTP program was then utilised to annotate these ORFs by identifying homologous proteins in the NCBI non‐redundant protein database. Subsequently, the DNA sequences encompassing 3 kb upstream and downstream of the annotated ORFs were subjected to gene prediction using the FGENESH+ program from the Softberry bioinformatics suite, with the homologous protein sequences identified from NCBI as the references. Following the gene prediction step, the homologous genes in the studied genomic regions across species were confirmed using the BLAST program.

## 3 Results

### 3.1 Molecular Classification of Thraustochytrid Strains Into Four PUFA Biosynthesis Pathway Utilisation Types

**FIGURE 1:**
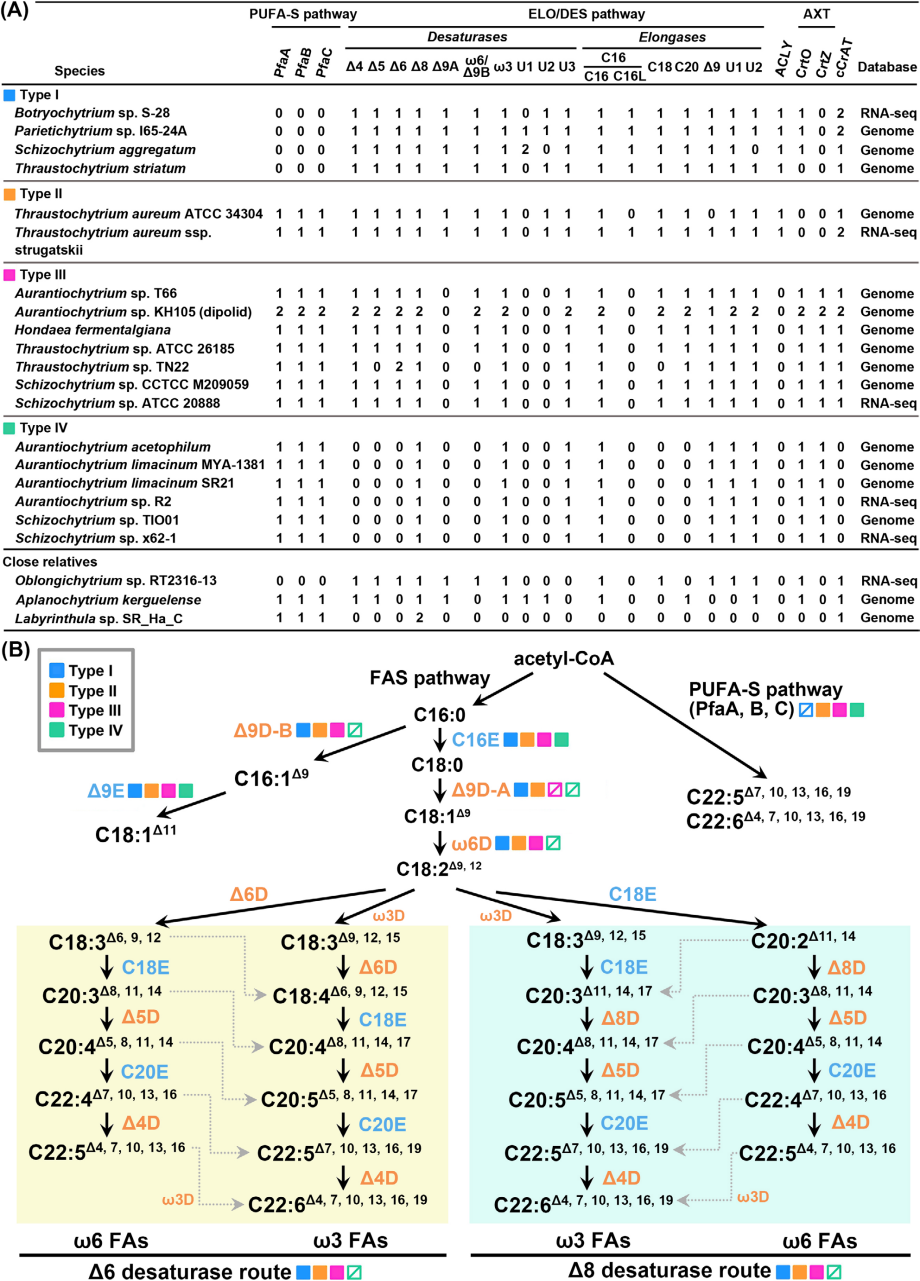
Overview of polyunsaturated fatty acids (PUFAs) biosynthesis pathways and the associated genes across four thraustochytrid lineages. (A) Distribution of genes involved in PUFA and astaxanthin biosynthesis pathways across the four thraustochytrid lineages. The genes depicted include three PUFA synthase genes (*PfaA*, *PfaB* and, *PfaC*), ten desaturase genes (Δ*4*, Δ*5*, Δ*6*, Δ*8*, Δ*9A*, *ω6/Δ9B*, *ω3*, *U1*, *U2*, and *U3*), six elongase genes (*C16*, *C18*, *C20*, Δ*9*, *U1*, and *U2*), one *ACLY* gene and two astaxanthin biosynthesis genes, *CrtZ* encoding β‐*CrtZ* and *CrtO* encoding β‐carotene ketolase. The genes were identified from either genomic or transcriptomic (RNA‐seq) databases. Close relatives refer to three species in the subclass Labyrinthulomycetes outside the family Thraustochytriaceae. (B) Illustration depicting the PUFA biosynthesis pathways across the four thraustochytrid lineages. Solid and slashed squares represent the presence and absence, respectively, of the corresponding genes in each PUFA biosynthetic type. Elongase is denoted in blue, and desaturase is denoted in orange. The Δ6DES route and Δ8DES route represent distinct biosynthetic processes utilising Δ6 desaturase and Δ8 desaturase enzymes, respectively, for the synthesis of ω3 and ω6 PUFAs. AXT indicates astaxanthin.

Contingent upon the strain and species, thraustochytrids exhibit variation in producing PUFAs, employing either the PUFA‐S or the elongase/desaturase (ELO/DES) pathway, or a combination thereof (Lippmeier et al. 2009; Nagano et al. 2011; Matsuda et al. 2012; Ishibashi et al. 2021). To investigate this variation and elucidate the distinct PUFA biosynthetic pathways across thraustochytrids, a comprehensive genome‐wide identification and comparative analysis of PUFA biosynthetic genes was conducted across 13 genomic and six RNA‐seq databases of 19 thraustochytrid strains belonging to six genera: six *Aurantiochytrium* (*Aurantiochytrium acetophilum*, *A. limacinum* ATCC MYA‐1381, *A. limacinum* SR21, *Aurantiochytrium* sp. T66, *Aurantiochytrium* sp. KH105 and *Aurantiochytrium* sp. R2), one *Botryochytrium* (*Botryochytrium* sp. S‐28), one *Hondaea* (*H. fermentalgiana*), one *Parietichytrium* (*Parietichytrium* sp. I65‐24A), five *Schizochytrium* ( *Schizochytrium aggregatum* , *Schizochytrium* sp. CCTCC M209059, *Schizochytrium* sp. ATCC 20888, *Schizochytrium* sp. TIO01 and *Schizochytrium* sp. x62‐1), and five *Thraustochytrium* (*Thraustochytrium striatum*, *Thraustochytrium aureum* ATCC 34304, *T. aureum* ssp. strugatskii, *Thraustochytrium* sp. ATCC 26185, *Thraustochytrium* sp. TN22; Figure 1A and Table 1). More detailed information has been provided in Tables S1 and S3 All strains, except *A*. sp. KH105, are haploid, with the latter being diploid. To ensure a comprehensive analysis, the study included three additional strains within Class *Labyrinthulomycetes* that are phylogenetically related to Order Thraustochytrida: *Oblongichytrium* sp. RT2316‐13 (Order Oblongichytrida) and *Aplanochytrium kerguelense* and *Labyrinthula* sp. SR_Ha_C (both from Order *Labyrinthulida*; Anderson and Cavalier‐Smith 2012; Bennett et al. 2017; Pan et al. 2017). Putative protein sequences were identified utilising the BLASTP, TBLASTN or HMMER programs, with protein sequences from *H. fermentalgiana* serving as queries owning to its well‐annotated genome. The presence of the individual pathway was determined by the existence of their distinctive PUFA biosynthetic genes, including fatty acid elongase and desaturase genes of the ELO/DES pathway, and the genes encoding the three subunits of PUFA‐S (*PfaA*, *PfaB* and *PfaC*) in the PUFA‐S pathway (Figure 1A,B). The nomenclature for elongase and desaturase gene homologues across the investigated thraustochytrid strains was based on the enzyme activities validated through previous experimental studies (Ohara et al. 2013; Ishibashi et al. 2021; Rau et al. 2021). Our results indicated that 15 thraustochytrid strains possess the PUFA‐S pathway, while only six thraustochytrid strains contain a complete ELO/DES pathway without any involved genes missing (Figure 1A). Among them, *T. aureum* was the sole species identified as possessing both pathways, consistent with previous reports (Matsuda et al. 2012).

Based on the PUFA biosynthesis pathway‐specific gene patterns in the investigated genomic and RNA‐seq databases, we categorised these 19 thraustochytrid strains into four lineages: Type I lineage that encompasses *B*. sp. S‐28, *P*. sp. I65‐24A, *S. aggregatum* and *T. striatum* exclusively features the ELO/DES pathway; Type II lineage that includes *T. aureum* ATCC 34304 and *T. aureum* ssp. strugatskii possesses both PUFA‐S and ELO/DES pathways; Type III lineage, which comprises *A*. sp. T66, *A*. sp. KH105 (diploids), *H. fermentalgiana*, *T*. sp. ATCC 26185, *T*. sp. TN22, *S*. sp. CCTCC M209059 and *S*. sp. ATCC 20888, possesses the PUFA‐S and an incomplete ELO/DES pathway, containing all desaturase genes except for a pivotal Δ9 desaturase (Δ*9DES‐A*) that produces oleic acid (C18:1, n‐9); and Type IV lineage that includes *A*. *acetophilum*, *A. limacinum* ATCC MYA‐1381, *A. limacinum* SR21, *A*. sp. R2, *S*. sp. TIO01 and *S*. sp. x62‐1 relies solely on the PUFA‐S pathway and exhibits even lower counts of desaturase and elongase genes when compared to other lineages (Figure 1A,B). Furthermore, the analysis of the three closely related species indicated that only *O*. sp. RT2316‐13 lacks the PUFA‐S pathway, *A. kerguelense* possesses the PUFA‐S pathway and an incomplete ELO/DES pathway, and *L*. sp. SR_Ha_C solely contains the PUFA‐S pathway.

### 3.2 Nine Distinct Fatty Acid Desaturases Were Identified Among the Investigated Thraustochytrid Strains, With the Sequence Encoding the Canonical Δ9 Desaturase Detected Exclusively in the Types I and II Strains

**FIGURE 2:**
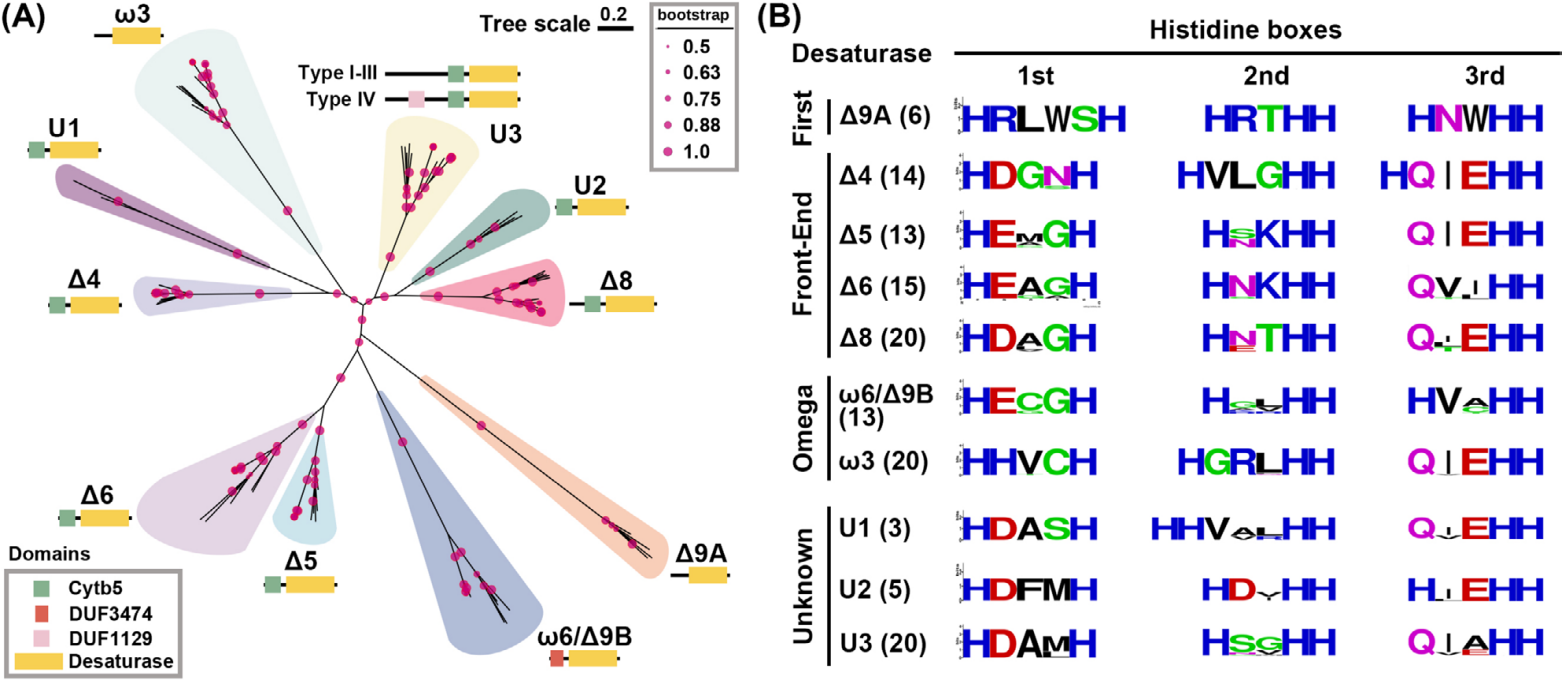
Phylogenetic analysis and conserved histidine boxes of desaturases in thraustochytrids. (A) Phylogenetic analysis of 129 desaturase protein sequences identified in 19 thraustochytrid strains. The unrooted phylogenetic tree was constructed using the neighbour‐joining (NJ) method with 1000 bootstrap replicates. The scale bar represents the number of amino acid substitutions per site. Bootstrap values exceeding 0.5 are indicated by magenta dots, with the dot size proportional to the bootstrap value magnitude. The predicted protein structure is illustrated beside each clustered desaturase group, with coloured rectangles denoting the predicted domains: Green indicates a cytochrome b5 domain (cytb5), orange indicates an unknown domain DUF3474, light pink indicates an unknown domain DUF1129, and yellow indicates the desaturase domain. (B) Sequence logo representation of the three conserved histidine boxes for each specific desaturase group, arranged in the order of desaturase subfamilies: First Desaturase, Front‐End Desaturase, Omega Desaturase and unknown. The sequence logos were generated using the WebLogo program based on multiple sequence alignments of the 129 desaturase protein sequences, performed using the MUSCLE algorithm. The number of desaturase sequences analysed for each desaturase group is indicated in quotation marks alongside the respective desaturase name.

Previous researches indicated that desaturases involved in PUFA biosynthesis contain a canonical desaturase motif encompassing three histidine boxes and can be categorised into three subfamilies based on their functional specificity: First Desaturase, Front‐End Desaturase, and Omega Desaturase (Hashimoto et al. 2008). At the N‐terminal region, both the First and Front‐End Desaturase subfamilies harbour a cytochrome b5 domain (Cytb5 domain: PF00173), while the Omega Desaturase subfamily contains a DUF3474 domain (Domain of UnKnown: PF11960; Mitchell and Martin 1995; Napier et al. 2003). The desaturase involved in the ELO/DES pathway includes one First Desaturase (Δ9DES‐A), four Front‐End Desaturases (Δ4, Δ5, Δ6 and Δ8 desaturases) and two Omega Desaturases (ω6/Δ9DES‐B and ω3 desaturase, Figure 1B). A recently characterised Omega Desaturase in *Aurantiochytrium* sp. T66 exhibits non‐canonical Δ9 desaturase activity, producing palmitoleic acid (C16:1, n‐7; Heggeset et al. 2019; Rau et al. 2021). Based on this bifunctional characteristic, we designated its homologous genes as ω6/Δ9DES‐B, while the homologues of canonical Δ9 desaturase that convert stearic acid (C18:0) to oleic acid (C18:1, n‐9) are termed Δ9DES‐A in this study. The identified desaturases in thraustochytrids were analysed using phylogenetic approaches and confirmed based on their distinctive sequence patterns (Figures 2A and S1). These desaturases were categorised into 10 distinct groups, seven of which (Δ4, Δ5, Δ6, Δ8, Δ9DES‐A, ω6/Δ9DES‐B and ω3 desaturases) correspond to previously identified and functionally characterised desaturases, while the remaining three groups, termed U1, U2 and U3 desaturases, represent uncharacterized desaturases in this study (Meesapyodsuk and Qiu 2016). All the Front‐End Desaturases identified in thraustochytrids (Δ4, Δ5, Δ6 and Δ8 desaturases), as well as the uncharacterized desaturases U1, U2 and U3, contain the cytb5 domain in the N‐terminal region. In contrast, the ω6/Δ9DES‐B desaturase harbours the DUF3474 domain (Figure 2A). Notably, the Δ9DES‐A (a First Desaturase) and ω3 desaturase in thraustochytrids lack these canonical N‐terminal domains. Moreover, the U3 desaturase exhibits an elongated N‐terminal region of approximately 500 amino acids upstream of the cytb5 domain, where no other conserved domains are predicted, except for the strains from the Type IV lineage, which possess a DUF1129 domain (PF06570) of unknown function. While genes or transcript sequences of desaturases were identified in all thraustochytrid lineages, Δ9DES‐A and U2 desaturase are exclusive to the Type I and Type II lineage strains, and the U1 desaturase is present solely in the two Type I lineage strains, *P*. sp. I65‐24A and *S. aggregatum* (Figure 1A). Conversely, strains of Type IV lineage possess only Δ8, ω3 and U3 desaturases. Furthermore, the phylogenetic analysis demonstrated that within each desaturase clade, protein sequences from strains of the same PUFA biosynthetic type cluster together, indicating their conserved features across the respective PUFA biosynthesis in each desaturase group (Figure S1). Hashimoto et al. previously documented the canonical histidine box configurations of desaturase and elongase enzymes through their conserved histidine regions (Hashimoto et al. 2008). The desaturase motif revealed distinct three‐box molecular signatures across different subfamilies: First Desaturases presented HRLWSH, HRXHH and HNXHH; Front‐End Desaturases displayed HDXGH, HXXXHH and QXEHH; and Omega Desaturases exhibited H(E/D)CGH, HXXHH and HVXHH. Sequence analysis of thraustochytrid desaturases confirmed the consistent three‐histidine‐box structure within the desaturase motif region, unveiling subtle yet significant variations from established canonical patterns. The Front‐End desaturases particularly demonstrated sequence divergence, with the second histidine box predominantly appearing as HXXHH and markedly deviating from previous descriptions. In contrast, the Δ4 desaturase group emerged as a unique molecular variant, preserving the canonical HXXXHH motif while exhibiting substantial divergence in its first and third box patterns. Equally noteworthy, the sequence patterns of all three histidine boxes in the ω3 desaturases differ markedly from established Omega Desaturase subfamily patterns. Moreover, the histidine boxes of the three uncharacterized desaturases, U1, U2 and U3, presented particularly complex profiles, with U1 and U3 showing significant variations in first and second histidine boxes, and U2 exhibiting comprehensive sequence divergence across all three boxes. These molecular variations created substantial ambiguity, preventing definitive categorization into existing desaturase groups.

### 3.3 A Total of Six Major Elongase Groups Were Identified in Thraustochytrids, With the C18 and C20 Elongases Being Absent in the Type IV Lineage

**FIGURE 3:**
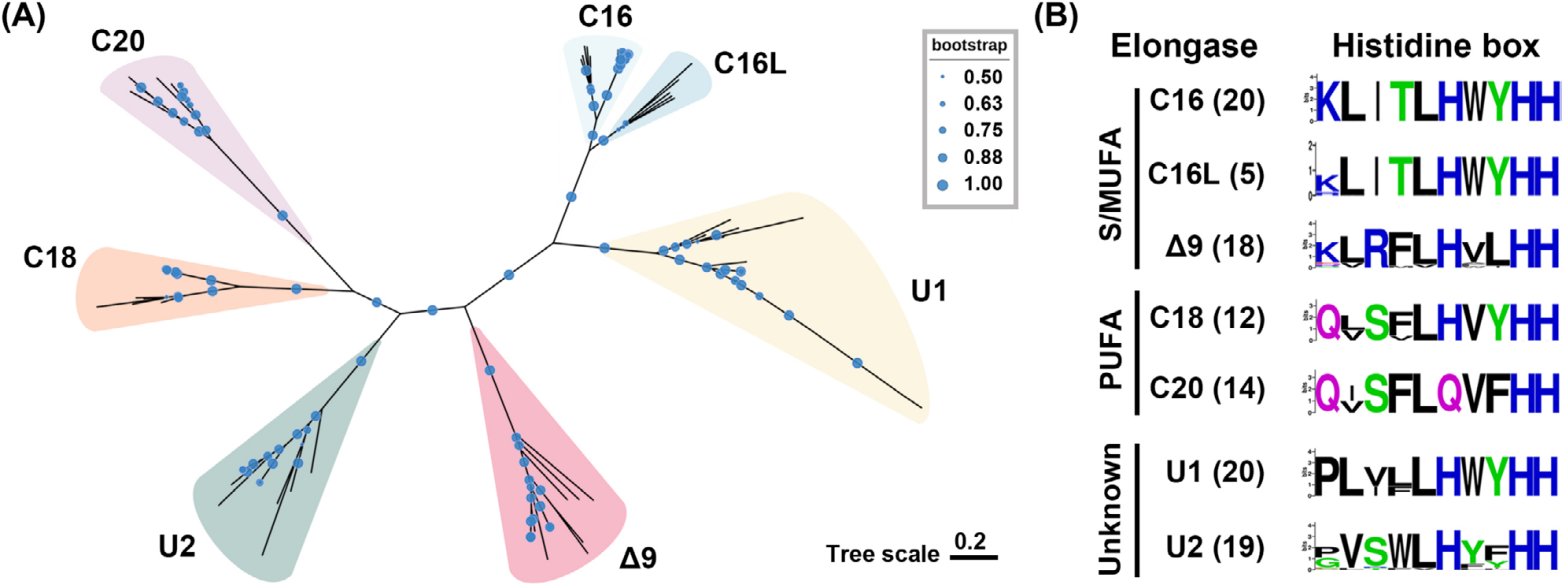
Phylogenetic analysis and conserved histidine boxes of elongases in thraustochytrids (A) An Unrooted phylogenetic tree constructed from 108 elongase protein sequences identified in 19 thraustochytrid strains. The tree was inferred using the neighbour‐joining (NJ) method with 1000 bootstrap replicates. The scale bar represents the number of amino acid substitutions per site. Bootstrap values exceeding 0.5 are marked with red dots, with dot size proportional to the bootstrap value magnitude. The seven elongase groups are labelled alongside their respective sequence clusters. (B) Sequence logo representation of the conserved elongase motif in different elongase groups, arranged in the order: Short/medium fatty acid elongase (S/MUFA) subfamily, PUFA elongase subfamily, and unknown elongase. The sole histidine box was extracted and visualised using the WebLogo online program. The numbers of elongase sequences analysed for each elongase group is indicated in parentheses following the desaturase group name.

The ELO/DES pathway involves three major elongases, including C16, C18 and C20 elongases, all of which contain only one elongase motif (PF01151) without any other characterised motifs. Thraustochytrid elongases can be categorised into six major groups, namely C16, C18, C20, Δ9, U1 and U2, alongside one minor group, termed C16‐like (C16L) elongase, based on phylogenetic tree analysis (Figure 3A). Akin to the desaturases, the elongase protein sequences from the same PUFA biosynthetic lineages clustered together within their respective clade in each elongase group (Figure S2). The C16L elongase shares high sequence homology with the C16 elongase (> 50% protein identity) and an identical histidine box within the elongase motif (Figure 3B). All major elongase groups were present in the Type I, II and III lineage strains, except for the sequences of C16L, which were found exclusively in the Types I and II lineage strains (Figure 1A). The Type IV lineage lacked the C18 and C20 elongases. Additionally, the two uncharacterised elongase groups, U1 and U2, were identified in *O*. sp. RT2316‐13 and all investigated thraustochytrid strains. However, no elongase sequences, including the U1 and U2 groups, were detected in the genome of *L*. sp. SR_Ha_C. Sequence analysis revealed that all elongases contain a canonical histidine box, HXXHH, except for the C20 elongase group, which harbours an atypical motif with the first histidine residue replaced by glutamine, QXXHH (fig. 3B; Hashimoto et al. 2008).

### 3.4 *ACLY* Was Identified in Types I and II Strains While *CrtZ* Is Present in Types III and IV Strains

ACLY plays a pivotal role in supplying the acetyl‐CoA pool for PUFA biosynthesis in the cytosol. However, several published thraustochytrid genomes lack homologous genes encoding known ACLY (Heggeset et al. 2019). To investigate whether this absence of ACLY corresponds to their PUFA biosynthetic types, a comprehensive search for ACLY homologous sequences was performed. The results revealed that ACLY homologues are present only in the Types I and II thraustochytrid strains but absent in the Types III and IV strains (Figure 1A). Additionally, previous studies have indicated that some thraustochytrid strains are valuable for producing carotenoids, including β‐carotene and xanthophylls (oxygenated carotenoids), which act as antioxidants against environmental stresses (Leyton et al. 2021; Liu et al. 2024). Consequently, two key enzymes involved in astaxanthin biosynthesis, β‐CrtZ and β‐carotene ketolase (CrtO), responsible for the synthesis of a series of xanthophylls, were examined (Iwasaka et al. 2018). It was observed that the homologous gene sequences encoding CrtO were identified in the Type I, III and IV lineages, while sequences encoding CrtZ were present only in the Types III and IV lineages, suggesting possible diversity in the xanthophylls composition among these lineages.

### 3.5 The Genomic Regions Harbouring the *Δ9DES‐A*, *ACLY* and PUFA Synthase Subunit Genes ( *PfaA* ‐ *PfaB* ) Exhibit a Conserved Syntenic Organisation Across the Examined Thraustochytrid Strains

**FIGURE 4:**
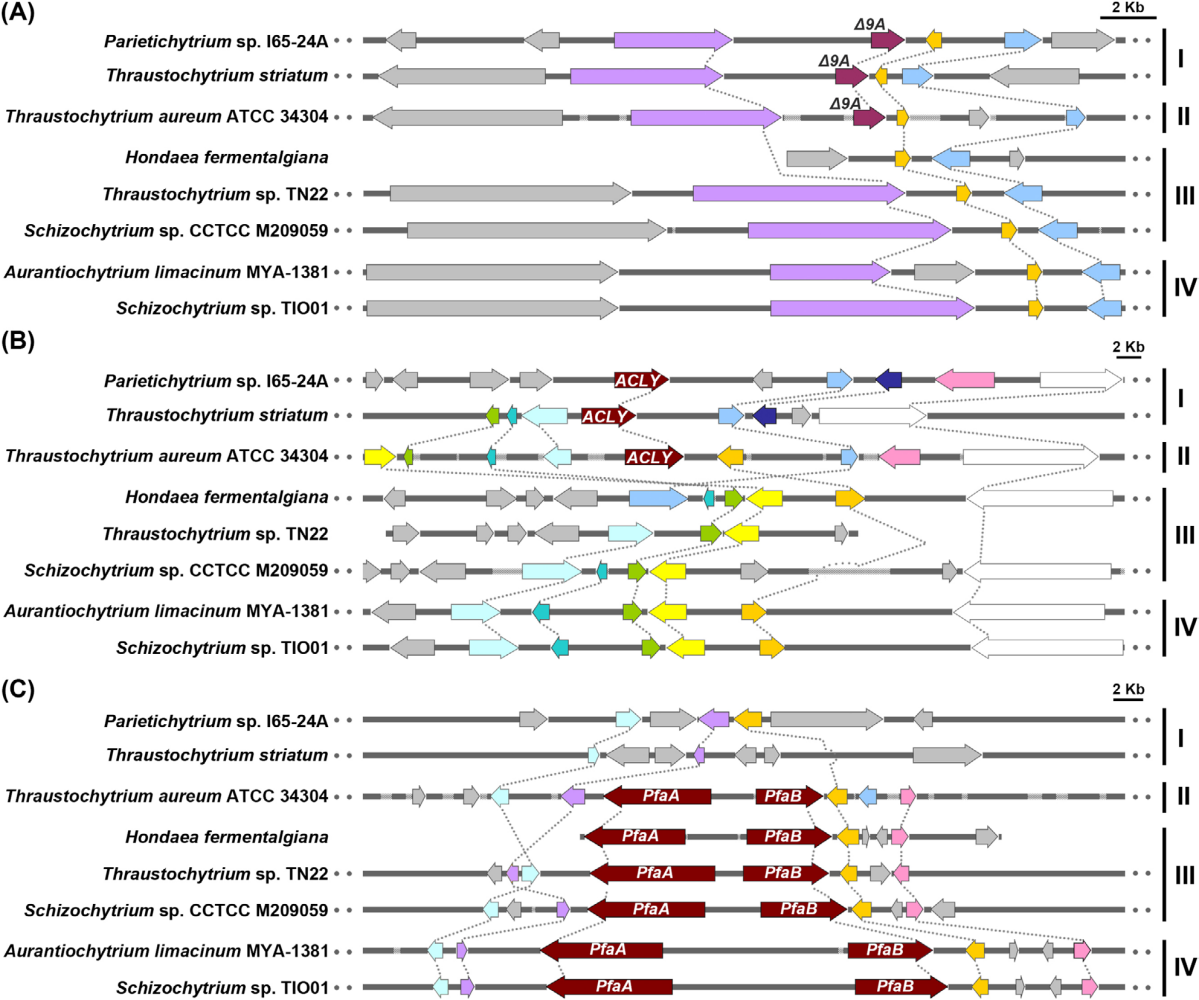
Syntenic analysis of genomic regions housing Δ*9DES‐A*, *ACLY*, and two PUFA‐S subunits *PfaA* and *PfaB* genes across thraustochytrid lineages. Syntenic analysis was performed using genome sequences from two strains in the Type I lineage (*Parietichytrium* sp. I65‐24A and *Thraustochytrium striatum*), one strain in the Type II lineage (*Thraustochytrium aureum*), three strains in the Type III lineage (*Hondaea fermentalgiana*, *Thraustochytrium* sp. TN22 and *Schizochytrium* sp. CCTCC M209059), and two strains in Type IV lineage (*Aurantiochytrium limacinum* and *Schizochytrium* sp. TIO01). Four upstream and four downstream genes adjacent to the target gene in *T. striatum* were used as queries to identify homologous genes for synteny analysis. In this figure, genes are represented as arrow‐shaped boxes (gene arrows), with the arrowhead indicating the direction of transcription. The target genes are represented by dark red gene arrows, while the homologous genes of the query genes are shown as arrows filled with their respective colours. Grey arrows represent non‐homologous query genes. (A) Syntenic analysis of the genomic region harbouring Δ*9DES‐A*, denoted as Δ*9A*. (B) Syntenic analysis of the genomic region harbouring *ACLY*. (C) Syntenic analysis of the genomic region harbouring the PUFA‐S subunits *PfaA* and *PfaB*. Bars indicate a length of 2 kb.

The presence or absence of Δ*9DES‐A*, *ACLY* and *PUFA‐S* gene homologues among the four PUFA biosynthetic types shows an organised pattern (Figure 1A). Specifically, Type II strains possess all three homologous genes, whereas Type I strains contain Δ*9DES‐A* and *ACLY* but lack *PUFA‐S* genes. In contrast, Types III and IV lineages contain PUFA‐S but not Δ9DES‐A desaturase and ACLY. To elucidate the evolutionary relationships of these genes, their syntenic organisation was investigated within the examined thraustochytrid strains. In our comparative analysis, we scrutinised the genomic locations of homologues for four upstream and four downstream genes flanking the target genes. Across the examined species, we observed syntenic conservation of one upstream and two downstream genes surrounding the Δ*9DES‐A* gene locus in thraustochytrid strains, as depicted in Figures 4A and S3. Furthermore, the genomic regions housing *ACLY* genes also exhibited synteny among thraustochytrids, although the arrangement of these syntenic genes in Types III and IV strains differs from that observed in Types I and II (Figures 4B and S4). The genes encoding Subunit‐A (*PfaA*) and Subunit‐B (*PfaB*) of the PUFA‐S complex were found adjacent to each other, forming the *PfaA*‐*PfaB* locus in the designated genomic regions of all three thraustochytrid lineages possessing the PUFA‐S pathway (Figures 4C and S5). Synteny analysis also revealed the conservation of this *PfaA*‐*PfaB* locus across thraustochytrid strains. Notably, the Type II thraustochytrid ( *T. aureum* ) harbours a greater number of orthologous genes within the region containing the *ACLY* gene compared to thraustochytrid strains of other types, while the other two syntenic regions show less pronounced differences (Figures 4 and S3–S5). Interestingly, closely related genomes, such as those of *A. kerguelense* and *L*. sp. SR_Ha_C do not exhibit synteny in the genomic regions encompassing genes involved in either ELO/DES or PUFA‐S pathways across any of the four thraustochytrid lineages.

### 3.6 The Mechanism of Acetyl‐CoA Replenishment in the Cytosol for PUFA Biosynthesis in ACLY‐Absent Lineages Remains to Be Determined

**FIGURE 5:**
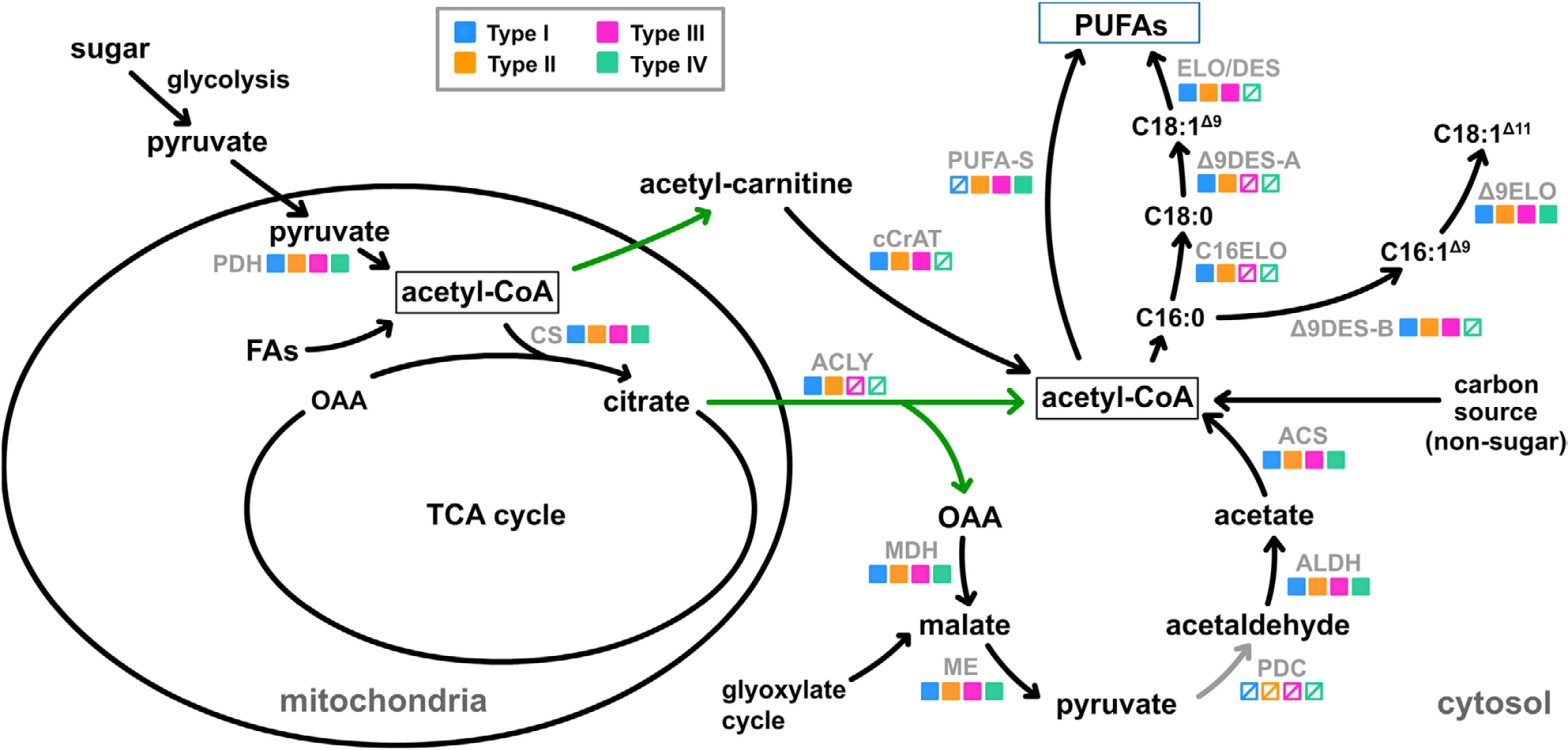
Schematic representation of the enzymatic pathways supplying cytosolic acetyl‐CoA for PUFA biosynthesis in four thraustochytrid lineages. This diagram summarises the critical steps involved in the intake of carbon sources that provide cytosolic acetyl‐CoA for PUFA biosynthesis. External carbon sources such as glucose undergo glycolysis to produce pyruvate, which is then transported to mitochondria for acetyl‐CoA synthesis in the matrix by PDH. The acetyl‐CoA is able to shuttle back to the cytosol to replenish the cytosolic acetyl‐CoA pool possibly through two primary routes: TCA cycle route, which is sequentially catalysed by CS and ACLY, or through direct shuttling as acetyl‐carnitine, catalysed by mitochondrial and cytosolic CrAT. Enzymes catalysing the designated steps are indicated in grey. The presence and absence of genes encoding individual enzymes in the four thraustochytrid lineages are represented by solid and slashed squares, respectively. Green arrows indicate routes of metabolite transport from mitochondria to the cytosol. Grey arrows represent catalytic steps absent in all thraustochytrid lineages. ACLY: ATP citrate lyase; ACS: acetyl‐CoA synthetase; ALDH: alcohol dehydrogenase; cCrAT: cytosolic carnitine acetyltransferase; CS: citrate synthase; DES: desaturase; ELO: elongase; ELODES: enzymes involved in the elongase/desaturase pathway; FAs: fatty acids; MDH: malate dehydrogenase; ME: malic enzyme; OAA: oxaloacetate; PDC: pyruvate decarboxylase complex; PDH: pyruvate dehydrogenase complex; PUFA‐S: Enzymes involved in the PUFA synthase pathway. TCA: tricarboxylic acid.

The absence of genes encoding ACLY in Types III and IV thraustochytrids renders the supplement of acetyl‐CoA in the cytosol for PUFAs biosynthesis unidentified. Acetyl‐CoA serves as the precursor for PUFA biosynthesis in both ELO/DES and PUFS‐S pathways. The lack of ACLY suggests the existence of alternative pathways for acetyl‐CoA replenishment. Pyruvate is a primary source for acetyl‐CoA and can undergo oxidative decarboxylation to yield a two‐carbon intermediate. In prokaryotic cells, the production of acetyl‐CoA is catalysed by pyruvate ferredoxin oxidoreductase (POR) and/or pyruvate formate lyase (PFL), while in eukaryotic cells, this process occurs via pyruvate dehydrogenase complex (PDH) in mitochondria and pyruvate decarboxylase complex (PDC) in the cytosol. However, sequences encoding either the prokaryotic enzymes or the eukaryotic PDC were not identified in any of the investigated thraustochytrids (Figure 5). It has been documented that the acetyl‐CoA shuttles via acetylcarnitine from mitochondria to cytosol, and the enzyme cytosolic carnitine acetyltransferase (cCrAT) catalyses the interconversion of acetyl‐CoA and acetylcarnitine (Schmalix and Bandlow 1993; Stemple et al. 1998). This pathway might serve as a crucial alternative route for acetyl‐CoA supply in thraustochytrids that lack both PDC and ACLY (fig. 5; Izzo et al. 2023). Consequently, after an extensive search, homologous genes encoding cytosolic *cCrAT* have been identified, with one or two copies present in Types I and II lineages, one copy in Type III lineages, but absent in Type IV lineage. All three closely related species also possess one gene copy per haploid genome size (Figure 1A).

### 3.7 Phylogenetic Analysis of 18S rRNA Gene Sequences Reveals Close Phylogenetic Relationships Among Thraustochytrids Within the Same PUFA Biosynthetic Types

**FIGURE 6:**
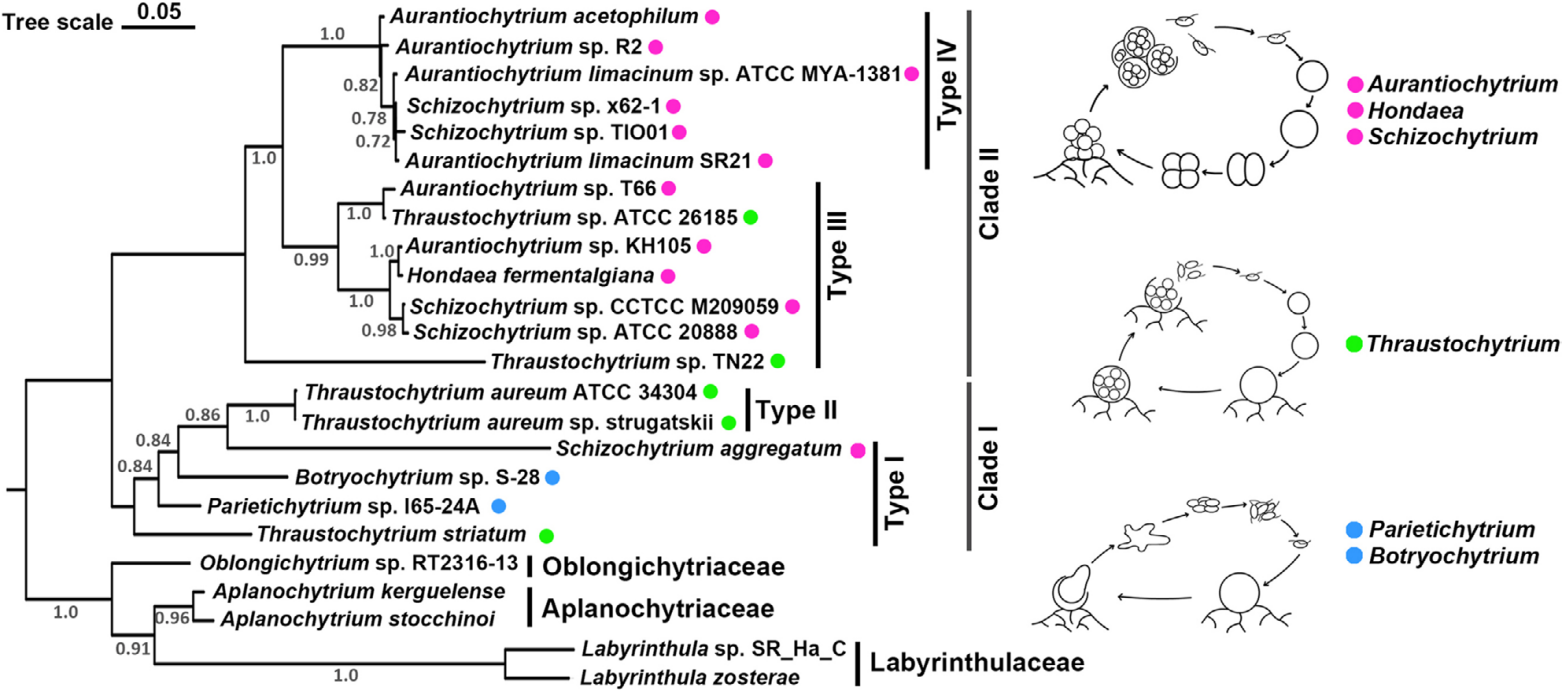
Phylogeny of the investigated thraustochytrid strains based on 18S ribosomal RNA (rRNA) gene sequences. The rooted tree depicts the phylogenetic relationships among the investigated thraustochytrids and their close relative species in taxonomy. The tree was constructed using 18S rRNA gene sequences from 19 thraustochytrid strains. One strain from the family Oblongichytriaceae, two strains from the family Aplanochytriaceae, and two strains from the family Labyrinthulaceae were employed as the outgroup. Sequences alignment was performed using the MAFFT program, and the phylogenetic tree was inferred using the Maximum Likelihood (ML) method with 1000 bootstrap replicates. The scale bar represents the number of amino acid substitutions per site. Three representative schematic life cycles exhibited by strains from six different thraustochytrid genera are depicted alongside the respective taxa and colour‐coded (Beakes et al. 2014).

In this study, 19 thraustochytrid strains were categorised into four distinct types based on their repertoire of PUFA biosynthetic genes. To investigate the evolutionary relationships among these types, we conducted a phylogenetic analysis using the 18S rRNA gene sequences from each strain. The Maximum Likelihood method was employed to reconstruct the phylogenetic tree, incorporating two more closely related species: *Aplanochytrium stocchinoi* and *Labyrinthula zosterae* (both from the Order *Labyrinthulida*), along with the previously mentioned relatives as the out‐group, to enhance the robustness of the analysis. The phylogenetic analysis revealed that thraustochytrids of the same PUFA biosynthetic type formed distinct monophyletic clades within the tree (Figure 6). Strains from Types I and II clustered together in a well‐supported clade (Clade I), while those from Types III and IV constituted separate monophyletic groups (Clade II). This topological pattern strongly suggests a correlation between the genetic machinery for PUFA biosynthesis and the evolutionary relationships among these thraustochytrid lineages.

## 4 Discussion

Thraustochytrids currently encompass 13 genera, with six examined in this study. Our analysis revealed four distinct PUFA biosynthetic types, suggesting a conserved yet diverse pattern of PUFA biosynthesis pathways across these organisms. Comparative gene analysis of PUFA biosynthesis pathways demonstrated that nearly all thraustochytrid strains possess either the polyunsaturated FAS (PUFA‐S) or a complete elongase/desaturase (ELO/DES) pathway, except for *T. aureum*, which harbours both. This suggests that either pathway alone suffices for PUFA production. However, the incomplete ELO/DES pathway found in Types III and IV thraustochytrids, which already possess PUFA‐S, implies its functional significance—potentially aiding in scavenging exogenous fatty acids or rescuing premature PUFA products. Moreover, the ELO/DES pathway demonstrates broader product diversity than the PUFA‐S pathway, generating unsaturated fatty acids ranging from C16 to C22, while PUFA‐S primarily produces docosapentaenoic acid (DPA, C22:5, n‐6) and DHA (C22:6, n‐3; Table S5). This broader range enables the ELO/DES pathway to synthesise fatty acids that cannot be synthesised by the PUFA‐S pathway, particularly C16:1, C18:1 and C18:2, which are crucial constituents of cell membrane. Type III thraustochytrids possess all the genes encoding enzymes involved in the ELO/DES pathway except Δ*9DES‐A*, which initiates the conventional pathway by producing oleic acid (C18:1, n‐9). This deficiency could impair subsequent reactions and membrane fatty acid production. Therefore, alternative mechanisms for C18:1 acquisition should exist, either through exogenous uptake or alternative desaturation processes. Previous studies demonstrated that the ω6 desaturase in *Aurantiochytrium* sp. T66 (Type III), designated here as ω6/Δ9DES‐B, exhibits non‐canonical Δ9 desaturase activity, producing palmitoleic acid (C16:1, n‐7; Heggeset et al. 2019; Rau et al. 2021), which could be subsequently converted to vaccenic acid (C18:1, n‐7) by Δ9 elongase (Ohara et al. 2013). Given that both Types III and IV thraustochytrids lack the conventional Δ*9DES‐A* but retain the Δ*9ELO* gene, further investigation of alternative desaturases that might exhibit non‐canonical Δ9 desaturase activity, as demonstrated in *A*. sp. T66, across these thraustochytrid lineages is warranted.

Glucose serves as the primary carbon source for PUFA biosynthesis in cultured thraustochytrids, where it is converted to pyruvate through glycolysis and subsequently to acetyl‐CoA in the mitochondria or cytosol. However, as shown in Figure 5, the absence of *PDC* and *ACLY* genes in Types III and IV thraustochytrids raises questions about their mechanism of acetyl‐CoA acquisition. Although an *ACLY* gene has been identified in one of the Type III strains, *Schizochytrium* sp. CCTCC 209059, it is a truncated form with only 422 residues in protein length (Han et al. 2020). Compared to the full‐length ACLY from Types I and II thraustochytrids that possess approximately 1000 residues, this truncated ACLY only contains the C‐terminal lyase domain but lacks the binding domains for ATP, citrate and CoA moieties in the N‐terminal region. Therefore, it is speculated that this truncated form is unable to recognise and catalyse the cleavage of citrate to produce acetyl‐CoA, and there might be an alternative pathway to replenish the acetyl‐CoA pool in the cytosol for fatty acid biosynthesis in these lineages. Some eukaryotic cells, such as fungi, can export acetyl‐CoA from mitochondria via CrAT shuttle (Schmalix and Bandlow 1993; Stemple et al. 1998). Nevertheless, homologous genes encoding cytosolic CrAT (cCrAT) were identified in the genomes of Type I–III thraustochytrids but were absent in Type IV lineages, leaving the source of acetyl‐CoA in Type IV thraustochytrids undetermined.

Beyond primary metabolism, thraustochytrids produce important secondary metabolites like carotenoids, which protect lipids from oxidative stress, with astaxanthin showing the highest antioxidant efficacy (Miki 1991; Ambati et al. 2014). The major carotenoids, typically associated with storage lipid droplets of thraustochytrids, are β‐carotene, canthaxanthin and astaxanthin (Liu et al. 2024). Our analysis revealed β‐carotene ketolase (*CrtO*) genes or transcripts in most thraustochytrids, except *T. striatum* (Type I) and all *T. aureum* strains (Type II), while β‐*CrtZ* was exclusive to Types III and IV lineages. This distribution suggests canthaxanthin as the primary carotenoid in most Type I strains, whereas zeaxanthin and/or astaxanthin likely predominate in Types III and IV. Consistent with these findings, astaxanthin is detected only in Types III and IV thraustochytrids, while Types I and II strains (*Botryochytrium*, *Parietichytrium*, *S. aggregatum* and *T. aureum* ) contain canthaxanthin (Yokoyama et al. 2007; Koopmann et al. 2023). Notably, *T. striatum* (Type I) produces astaxanthin despite lacking both *CrtO* and *CrtZ* genes (Xiao et al. 2019; Koopmann et al. 2023), suggesting either an uncharacterised biosynthetic mechanism for astaxanthin production or incomplete genomic coverage that may have missed these critical genes.

Phylogenetic analysis of 18S rRNA sequences (Figure 6) revealed two major thraustochytrid clades: Clade I (Types I and II) and Clade II (Types III and IV), reflecting close evolutionary relationships within each clade. This aligns with their gene profiles, as Types I and II harbour Δ*9DES‐A* and *ACLY* genes, while *CrtZ* is exclusive to Types III and IV. The presence of both the PUFA‐S and complete ELO/DES pathways in Type II suggests its intermediate evolutionary position between the two clades. Gene synteny analysis revealed that genomic regions harbouring Δ*9DES‐A*, *ACLY* or *PfaA/PfaB* genes are syntenic across strains of the four thraustochytrid lineages, suggesting a common evolutionary origin for these genes. Notably, Type II thraustochytrids share more syntenic regions with the other three types compared to the synteny observed between the other types, further supporting the hypothesis that Type II may occupy an intermediate position in the evolutionary relationships among the four lineages.

**FIGURE 7:**
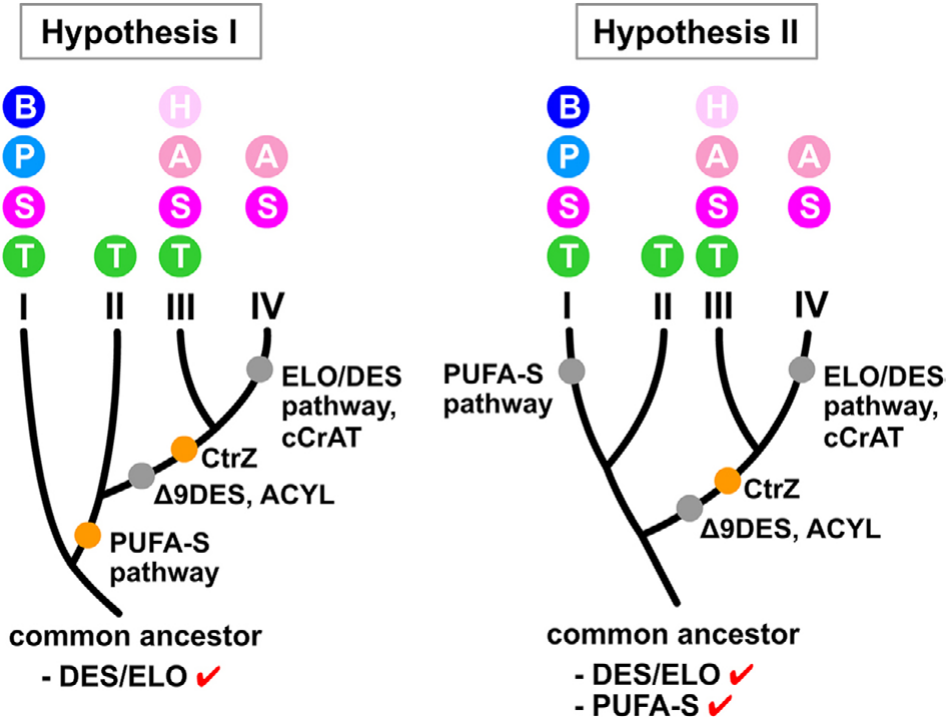
Proposed evolutionary trajectories of the four thraustochytrid lineages based on PUFA biosynthesis pathways. Two potential evolutionary pathways are depicted for the four PUFA biosynthetic lineages: Types I, II, III and IV. Orange and grey dots, respectively, represent the presence and absence of genes or the PUFA biosynthesis pathways. The genera associated with each lineage are represented by their initial letter, with colour coding corresponding to the life cycle illustrations in Figure 6. A: *Aurantiochytrium*, B: *Botryochytrium*, H: *Hondaea*, P: *Parietichytrium*, S: *Schizochytrium*, T: *Thraustochytrium*.

The ELO/DES pathway is considered ancestral and conserved in eukaryotes, whereas PUFA‐S genes were presumably acquired from marine bacteria through horizontal gene transfer (Metz et al. 2001). This suggests two possible evolutionary scenarios for thraustochytrids. In the first scenario (Figure 7, hypothesis I), their common ancestor possessed only the ELO/DES pathway, with Type II thraustochytrids emerging from Type I through PUFA‐S pathway acquisition. The sequential loss of ELO/DES pathway components, such as Δ*9DES‐A* and *ACLY*, led to the emergence of Types III and IV lineages, with Type IV further losing both the complete ELO/DES pathway and the *cCrAT* gene. Comparative genomic analysis of three phylogenetically related taxa outside Thraustochytrida provides additional evolutionary context. Both *A. kerguelense* and *Labyrinthula* sp. SR_Ha_C (Order Labyrinthulida) possess the PUFA‐S pathway, while *A. kerguelense* and *Oblongichytrium* sp. RT2316‐13 (Order Oblongichytrida) contain the ELO/DES pathway. The co‐occurrence of both pathways in *A. kerguelense* and their distribution across these investigated orders suggests that both PUFA‐S and ELO/DES pathways might have been present in the common ancestor of Orders Labyrinthulida, Oblongichytrida and Thraustochytrida. This leads to an alternative hypothesis (Figure 7, hypothesis II): the ancestral thraustochytrid possessed both pathways. Under this scenario, a subsequent selective loss is postulated, with Type I thraustochytrids losing the PUFA‐S pathway while Types III and IV lost the ELO/DES pathway. Additionally, the *CrtZ* gene's absence in all three relatives and Types I and II thraustochytrids, but presence in Types III and IV, suggests its acquisition during the evolution of the latter lineages.

Our phylogenetic analyses of desaturases, elongases, and 18S rRNA sequences consistently showed that strains with similar PUFA biosynthetic types clustered together, indicating close evolutionary relationships within each biosynthetic group. This PUFA biosynthesis‐based classification was further supported by their distinct life cycle characteristics. As depicted in Figure 6, Type I members, including strains of *Botryochytrium* and *Parietichytrium*, share the distinctive characteristic of releasing free‐living amoeboid cells from the parental thallus. Notably, this amoeboid cell feature was also documented in *T. striatum* , a *Thraustochytrium* strain within the Type I lineage (Schneider 1967). The Type II lineage was represented exclusively by the *Thraustochytrium* genus characterised by zoospore release from sporangia (Morabito et al. 2019). Type III and IV thraustochytrids predominantly exhibit similar life cycles, featuring binary division producing mononucleated cell clusters, with limited exceptions of two *Thraustochytrium* strains (Figure 6). Overall, these observations suggest potential evolutionary correlations between metabolic capabilities and developmental strategies. Nevertheless, despite these coherent patterns between the clustered PUFA biosynthetic types and life cycle characteristics, we observed complex and often contradictory taxonomic relationships across our analyses. We found that while the Type II lineage consists of a single genus, Types I, III and IV comprise 2–4 genera each. Specifically, three genera—*Schizochytrium*, *Thraustochytrium*, and *Aurantiochytrium*—individually occur across multiple PUFA synthesis lineages. This inconsistency between current genus‐level classification and PUFA pathway‐based categorization diminishes the correlation between thraustochytrid taxonomy and their metabolic and developmental characteristics. Furthermore, the distribution of *Schizochytrium* and *Thraustochytrium* strains across both Type I and Type III PUFA biosynthetic lineages, despite their distinct developmental and morphological traits, suggests either parallel evolution of these pathways or underlying taxonomic misclassification.

These observed taxonomic complexities align with previously documented polyphyletic relationships within thraustochytrid taxa (Jakobsen et al. 2007; Wang et al. 2024), reflecting persistent challenges in classification that hinder accurate evolutionary reconstructions and species identification. The historical use of *Thraustochytrium* as a ‘wastebasket taxon’ for species lacking clear taxonomic placement has further complicated these challenges, often creating discrepancies between molecular‐based relationships and formal taxonomic classifications. Recent studies have attempted to resolve these taxonomic inconsistencies through polyphasic approaches that integrate advanced morphological, biochemical, and phylogenomic analyses, leading to multiple reclassification events, as exemplified by the redesignation of *Schizochytrium limacinum* to *A. limacinum* or the establishment of new genera such as *Hondaea* and *Monorhizochytrium* (Yokoyama and Honda 2007; Doi and Honda 2017; Dellero, Cagnac, et al. 2018). Significantly, adopting the taxonomic revision proposed by Dellero, Cagnac, et al. (2018) could help resolve the phylogenetic inconsistency observed in our study, particularly in the Type III lineage. Based on robustly supported super clades from 18S rDNA sequence analysis coupled with both morphological and biochemical characteristics in that report, several Type III strains in our study—*Aurantiochytrium* sp. T66, *Aurantiochytrium* sp. KH105, *Thraustochytrium* sp. ATCC 26185, *Schizochytrium* sp. CCTCC M209059 and *Schizochytrium* sp. ATCC 20888—should be repositioned into *Hondaea* gen. nov. (Dellero, Cagnac, et al. 2018). When applying this reclassification framework, *Hondaea* emerges as the predominant genus within the Type III lineage, while *Aurantiochytrium* strains are exclusively confined to the Type IV clade. Given its phylogenetic position between Type I/II (clade I) and Type IV, *Hondaea* likely represents an evolutionarily significant genus that diverged earlier than the Type IV lineage genera *Aurantiochytrium* and *Schizochytrium*. This taxonomic reorganisation, while not definitive, resolves previous inconsistencies between genus assignments and lifecycle characteristics in relation to our PUFA biosynthetic types (Figure 6), and suggests a more coherent evolutionary history for these organisms.

Based on these findings, future reassignment of certain strains into different genera may be warranted. Our study demonstrates that integrating metabolic signatures, particularly PUFA biosynthesis pathway‐specific gene patterns, with polyphasic taxonomy provides new perspectives for understanding evolutionary relationships among thraustochytrids. This approach may help resolve current taxonomic ambiguities and facilitate more accurate prediction of metabolic capabilities based on phylogenetic position, with significant implications for both ecological research and biotechnological applications.

## Author Contributions

**Sou‐Yu Cheng:** conceptualization, formal analysis, investigation, validation, visualization, writing – original draft, writing – review and editing. **Yi‐Jing Chen:** formal analysis, data curation, methodology, software, visualization, writing – review and editing. **Hsiu‐Chin Lin:** writing – review and editing, resources. **Hsin‐Yang Chang:** writing – review and editing. **Ming‐Der Huang:** conceptualization, data curation, formal analysis, funding acquisition, investigation, methodology, project administration, resources, software, supervision, validation, visualization, writing – original draft, writing – review and editing.

## Ethics Statement

The authors have nothing to report.

## Conflicts of Interest

The authors declare no conflicts of interest.

## Supporting information


Figure S1.
Figure S2.
Figure S3.
Figure S4.
Figure S5.

Table S1.
Table S2.
Table S3.
Table S4.
Table S5.

## Data Availability

All database sources are cited within the article. The complete set of sequences used in this study is provided in the Supporting Information. All Supporting Information, including Figures S1–S5 and Tables S1–S5, have been deposited in Zenodo and are publicly available at: https://doi.org/10.5281/zenodo.15063618.
