# Supplementary material for: Genetic Analysis of Polyunsaturated Fatty Acids Biosynthesis Pathway Determines Four Distinct Thraustochytrid Types
Source: Environ Microbiol. 2025 Mar 28;27(4):e70090. doi: 10.1111/1462-2920.70090 (PMC11951076; doi:10.1111/1462-2920.70090)
Supplement: Supplementary file 1 — Figure S1. Figure S2. Figure S3. Figure S4. Figure S5. [file EMI-27-e70090-s001.pdf]

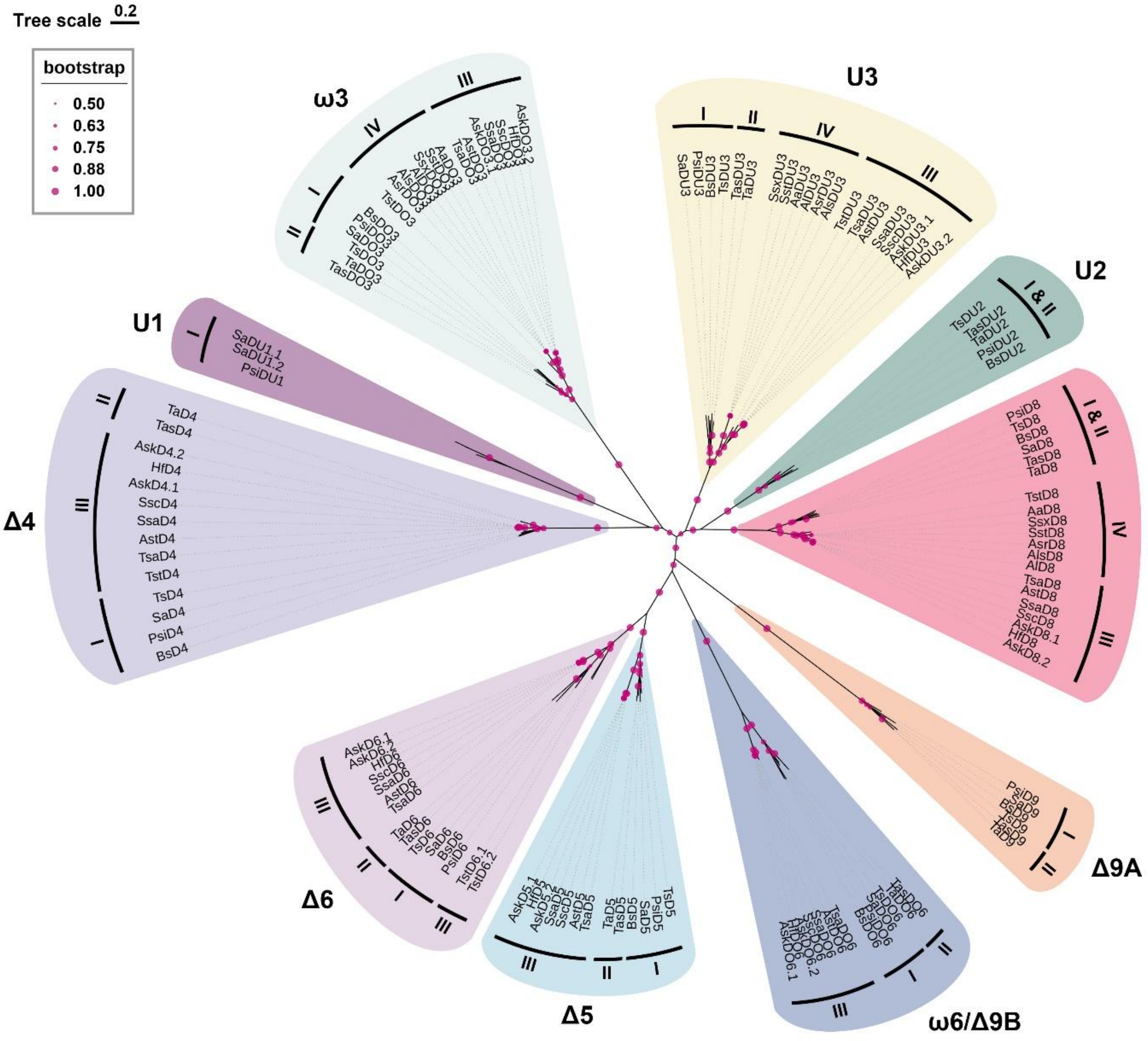

Supplementary Figure S1. Unrooted phylogenetic tree of 129 desaturase protein sequences with individual sequence names and their PUFA biosynthesis types. This tree is the same one as the tree in Figure 2A. The desaturase protein sequences, including Δ4, Δ5, Δ6, Δ8, Δ9A, ω3, ω6/Δ9B, and uncharacterized desaturases (U1, U2 and U3), were aligned using the MAFFT program. The phylogenetic tree was constructed using the Neighbor-Joining (NJ) method and supported by 1,000 bootstrap replicates. The dot size is proportional to the bootstrap value ranging from 0.5 to 1. The tree scale indicates the number of amino acid substitution per site. The sequence name code for each desaturase consists of the strain initials (corresponding to Supplementary Table S2), followed by "D" for desaturase, and either number for Δ desaturases, "O" for ω desaturases, or "U" for Uncharacterized desaturases.

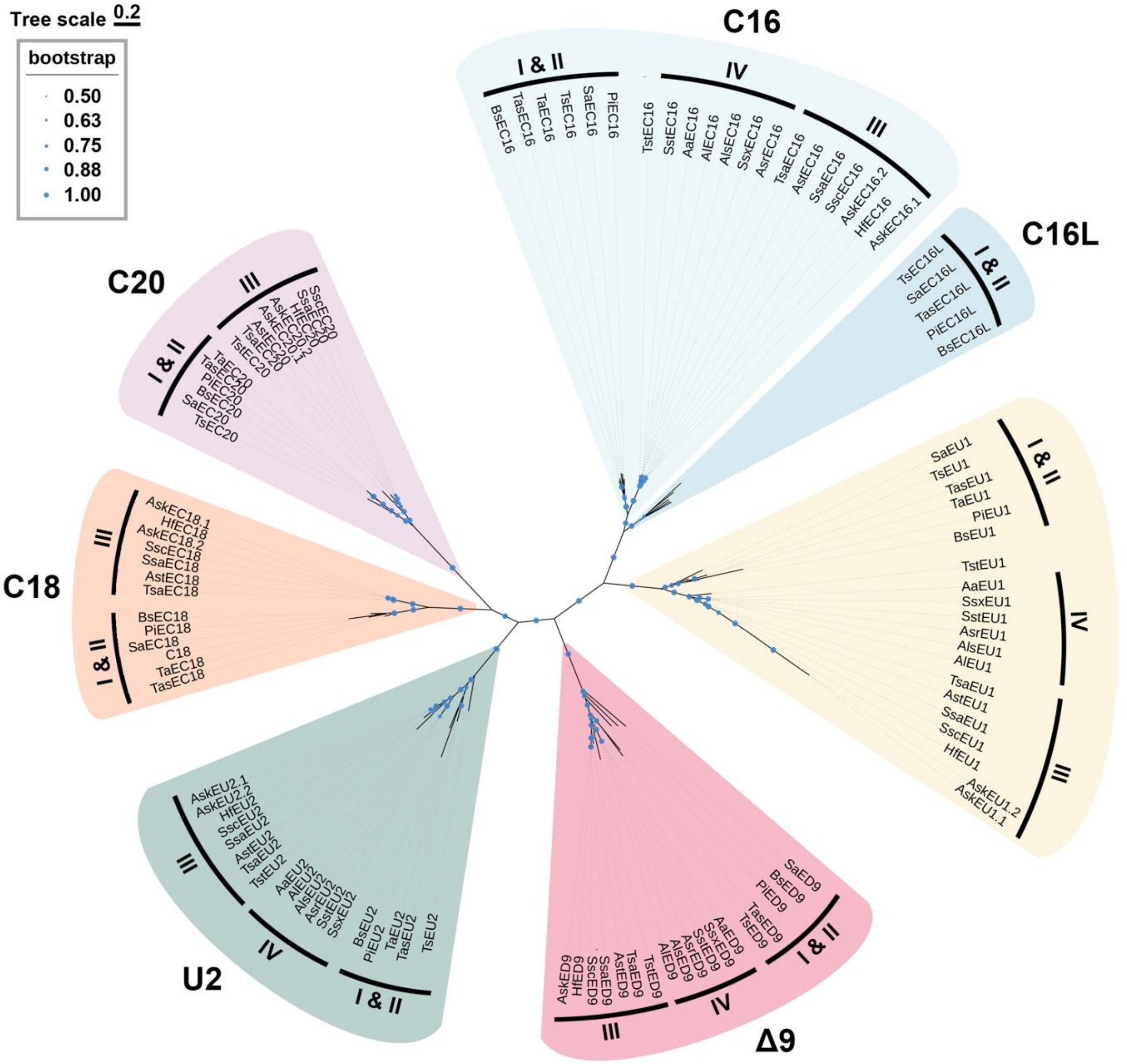

Supplementary Figure S2. Unrooted phylogenetic tree of 108 elongase protein sequences with individual sequence names and their PUFA biosynthesis types. The elongase protein sequences, including C16, C16L, C18, C20, Δ9, and uncharacterized elongases (U1, and U2) were aligned using the MAFFT program. The phylogenetic tree was constructed using the Neighbor-Join (NJ) method and supported by 1,000 bootstrap replicates. The dot size is proportional to the bootstrap values ranging from 0.5 to 1. The tree scale indicates the number of amino acid substitution per site. The sequence name code for each elongase consists of the strains initials (based on Supplementary Table S1), followed by "E" for elongase, and either "D9" for Δ9 elongase, or U" for Uncharacterized elongase.

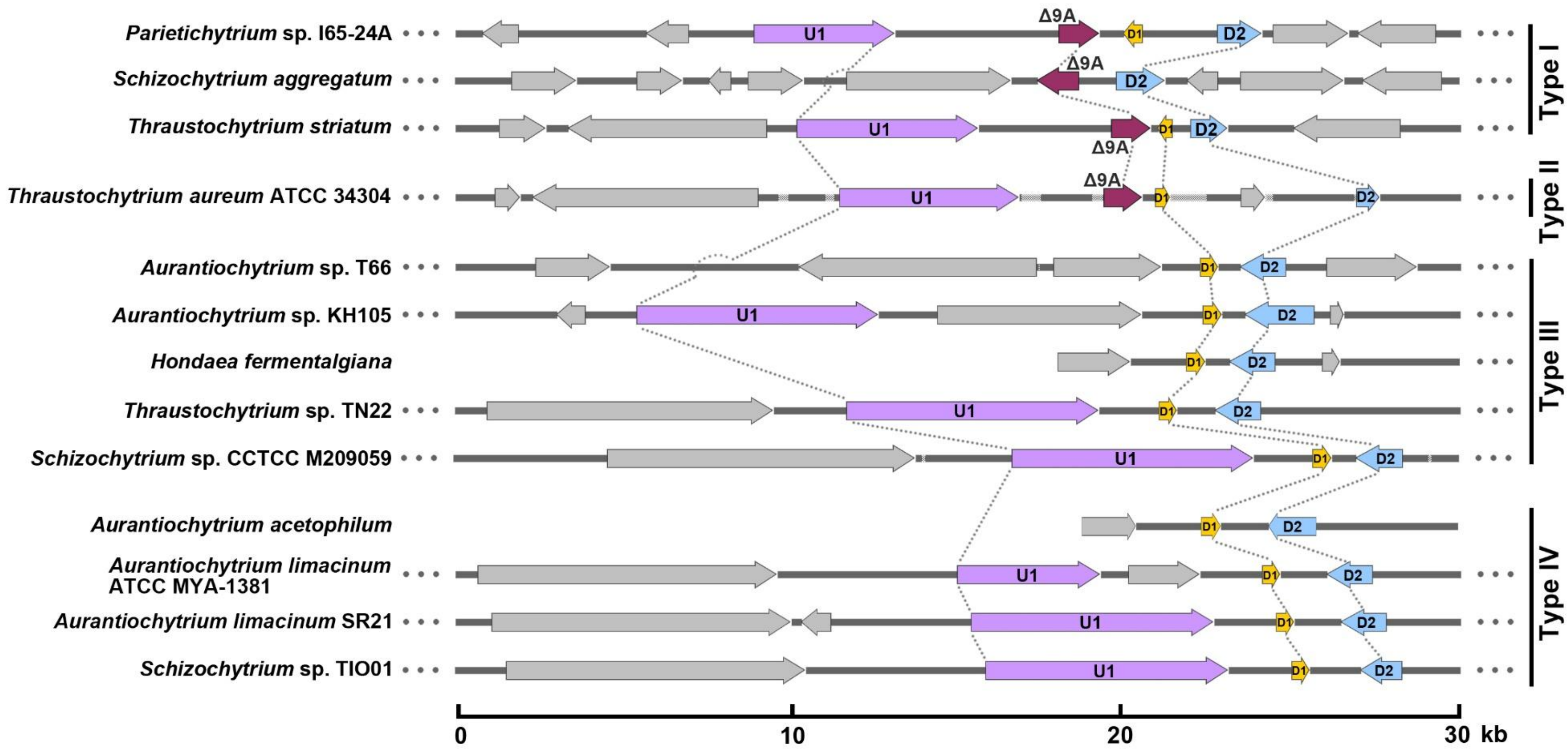

Supplementary Figure S3. Syntenic analysis of genomic regions containing the Δ9 desaturase (*Δ9DES-A*) gene across thraustochytrid lineages. The analysis was conducted within a 30 kb region encompassing both upstream and downstream areas of the *Δ9DES-A* gene (*Δ9A*). Homologous genes upstream (U1) and downstream genes (D1 and D2) of *Δ9DES* in the examined thraustochytrids are represented by colored arrow boxes and connected with dotted lines. Grey gene arrows represent genes lacking homology among the four types of thraustochytrids analyzed.

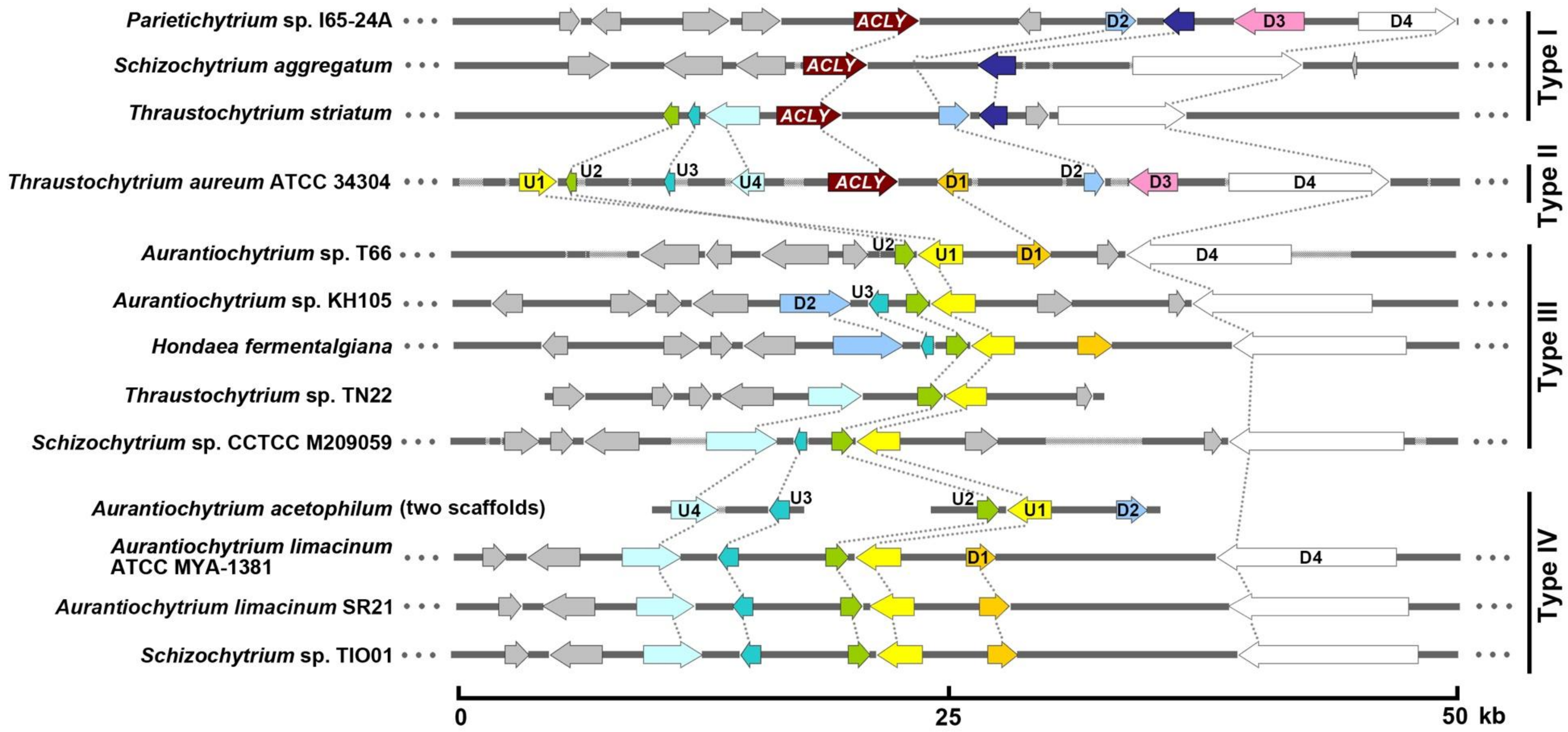

Supplementary Figure S4. Syntenic analysis of genomic regions containing the ATP-citrate lyase (*ACLY*) gene across thraustochytrid lineages. The analysis was performed within a 50 kb region encompassing both upstream and downstream area of the *ACLY* gene. Homologous genes upstream (U1, U2, U3, and U4) and downstream genes (D1, D2, and D3) of *ACLY* in the examined thraustochytrids are represented by colored arrow boxes and connected with dotted lines. Grey gene arrows represent genes lacking homology among the four types of thraustochytrids analyzed.

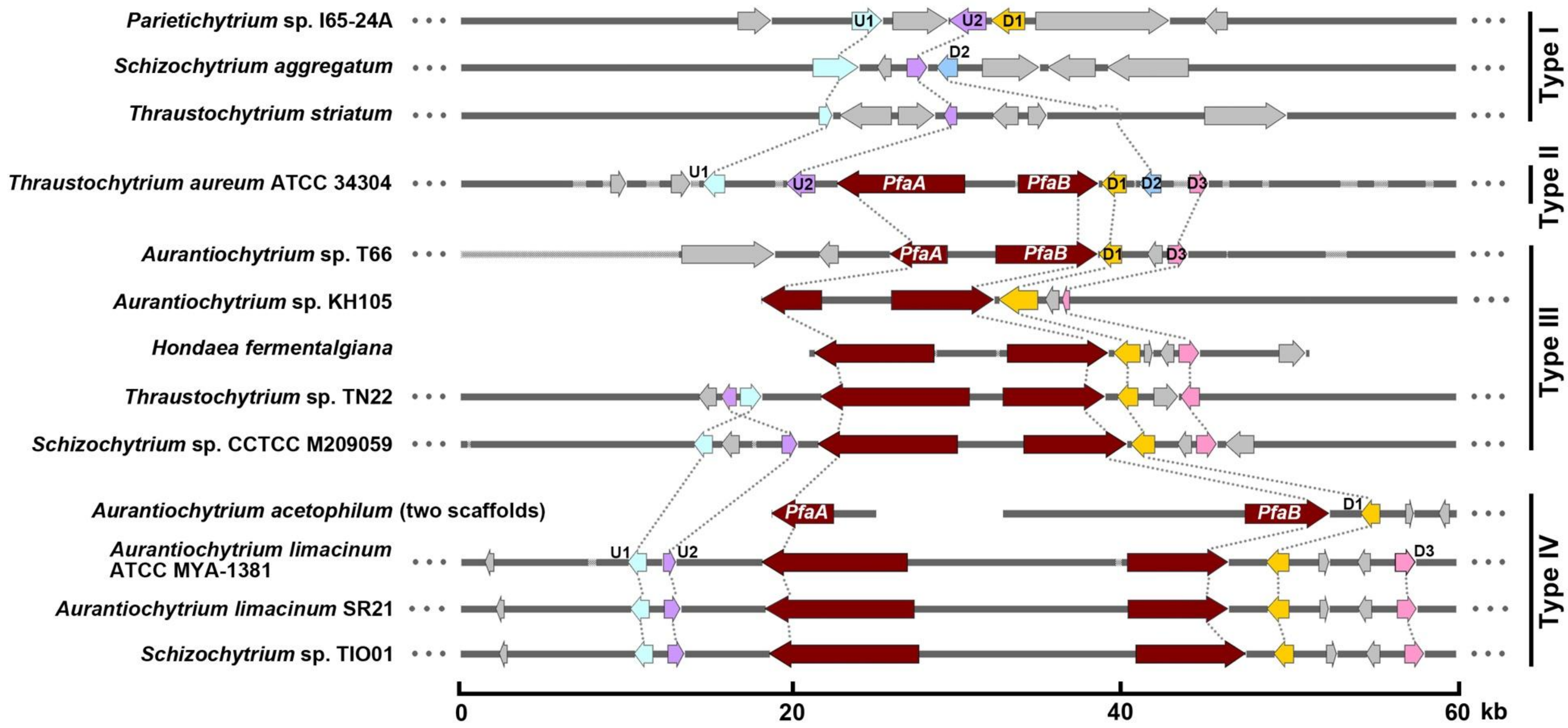

Supplementary Figure S5. Syntenic analysis of genomic regions containing the PUFA synthase subunit genes (*PfaA* and *PfaB*) gene across thraustochytrid lineages. The analysis was performed within a 60 kb region encompassing both the upstream and downstream area of the *PfaA* and *PfaB* genes. Homologous genes upstream (U1 and U2) and downstream genes (D1, D2, and D3) of *PfaA* and *PfaB* in the examined thraustochytrids are represented by colored arrow boxes and connected with dotted lines. Grey gene arrows represent genes lacking homology among the four types of thraustochytrids analyzed.
